# Changes in Upper Airway Anatomy and Apnea–Hypopnea Index in Patients with Obstructive Sleep Apnea Undergoing Bariatric Surgery: A Pilot Study

**DOI:** 10.3390/jcm15114038

**Published:** 2026-05-23

**Authors:** Maskani Nithya, Renuka Titiyal, Anuj Ajayababu, Bhavesh Mohan Lal, Akanksha Sinha, Surabhi Vyas, Sandeep Aggarwal, Andrew Wiemken, Richard J. Schwab, Brandon Nokes, Atul Malhotra, Sanjeev Sinha

**Affiliations:** 1 Department of Medicine, All India Institute of Medical Sciences, New Delhi 110029, India; 2 All India Institute of Medical Sciences, New Delhi 110029, India; 3 Department of Radiodiagnosis, All India Institute of Medical Sciences, New Delhi 110029, India; 4 Department of Surgical Disciplines, All India Institute of Medical Sciences, New Delhi 110029, India; 5 Division of Sleep Medicine, University of Pennsylvania Perelman School of Medicine, Philadelphia, PA 19104, USA; 6 Pulmonary, Critical Care and Sleep Medicine, UC San Diego School of Medicine, San Diego, CA 92121, USA

**Keywords:** obstructive sleep apnea—OSA, upper airway anatomy, magnetic resonance imaging, volumetric airway changes, soft tissue volumes, bariatric surgery, weight loss, correlation

## Abstract

**Background:** Obstructive sleep apnea (OSA) is common in individuals with obesity, largely due to increased soft tissue causing upper airway narrowing. However, mechanisms of OSA improvement following weight loss are incompletely understood, particularly in Asian population, where craniofacial and soft tissue characteristics differ. This study aimed to evaluate changes in upper airway anatomy before and six months after bariatric surgery. **Methods:** We prospectively evaluated Indian obese patients with OSA undergoing bariatric surgery. Magnetic resonance imaging (MRI) and polysomnography were performed at baseline and six months post-surgery to assess volumetric changes in upper airway structures and the apnea–hypopnoea index (AHI), respectively. Correlations between MRI-derived structural changes, weight loss, and AHI were also analyzed. **Results:** Ten obese patients with OSA were included. Bariatric surgery resulted in significant reductions in body weight, body mass index (BMI), Epworth Sleepiness Scale score, and AHI (*p* < 0.05). MRI demonstrated a significant reduction in overall pharyngeal soft tissue volume, soft palate, pterygoid and parapharyngeal fat pad volume and tongue fat fraction. However, no significant changes were observed in total upper airway volume, retroglossal and retropalatal airway volume. Furthermore, no significant correlation was noted between changes in upper airway anatomy and post-operative changes in AHI or body weight. **Conclusions:** Bariatric surgery was associated with significant weight loss and improvement in OSA severity, accompanied by reduction in soft tissue volumes without significant increase in airway volume in this pilot study. These findings suggest a possible role of factors other than structural airway changes in the observed improvement in OSA following bariatric surgery.

## 1. Introduction

Obstructive Sleep Apnea (OSA) is a sleep disorder expressed as recurrent nocturnal episodes of upper airway obstruction during sleep. OSA results in intermittent oxygen desaturation, sympathetic overdrive, fragmented sleep, and poor sleep quality, leading to excessive daytime sleepiness, reduced functional capacity, and substantially increased risk of cardiovascular events [1,2]. Globally, an estimated 936 million individuals aged 30 to 69 years are affected by OSA, with approximately 425 million cases classified as severe (95% CI: 903–970 million) [3]. In India, the prevalence of OSA is estimated to be approximately 11%, more frequent in males (13%) than in females (5%) [4].

OSA is more common among those with obesity compared to lean individuals. Narrowing of the upper airway owing to increased size of the pharyngeal soft tissues is postulated to be the cause for the high prevalence of OSA in people with obesity. Several studies conducted outside Asia have reported that individuals with OSA, compared to those without OSA, tend to exhibit increased volume of the tongue, tongue fat, soft palate, and lateral pharyngeal walls [5,6,7,8,9,10]. However, upper airway imaging studies involving Asian populations have produced variable and inconclusive results [11,12,13,14,15,16]. Bariatric surgery has increasingly been recognized as an effective therapeutic option for individuals with OSA who also have moderate to severe obesity. By reducing excess body fat, this procedure may help mitigate anatomical factors contributing to OSA, including those related to the pharynx, thoracic cavity, and visceral regions. Studies examining changes in upper airway structures following weight loss or bariatric surgery have also reported mixed results, with one study demonstrating a change, while the other did not [9,10]. There is a paucity of literature related to the effect of weight loss on changes in dimensions of various upper airway structures, and whether these changes in airway structures predict improvement in OSA. Thus, we evaluated the upper airway structures using magnetic resonance imaging (MRI) before and 6 months after bariatric surgery to examine the changes in these pharyngeal soft tissue volumes. Also, we performed polysomnography (PSG) before and after bariatric surgery to examine changes in apnea–hypopnea index (AHI). We also assessed the correlation between the change in volumes of the upper airway structures, weight loss, and AHI using standard statistical tools.

The primary objective of the study was to evaluate the changes in the volumes of upper airway soft tissue structures in obese patients with OSA through MRI of upper airway at baseline and six months post bariatric surgery. The secondary objective was to find the correlation between change in dimensions/volumes of upper airway, AHI, and weight loss following bariatric surgery in obese patients with OSA.

## 2. Materials and Methods

### 2.1. Study Design and Participants

This was a prospective observational study conducted at a tertiary referral center, All India Institute of Medical Sciences (AIIMS), New Delhi, India between August 2021 to June 2024. The study obtained ethics approval from the Institute Ethics Committee prior to study initiation (IECPG 385/23.06.2021, OT-8/27.04.2022). Adult patients aged ≥ 18 years with a body mass index (BMI) > 35 kg/m^2^ who were newly diagnosed with OSA based on polysomnography (PSG) were enrolled after obtaining informed consent. Participants with chronic obstructive pulmonary disease, congestive heart failure, any neck lesion or mass, a history of recent neurologic event, and pregnant females were excluded. Patients already on continuous positive airway pressure (CPAP) therapy or with other co-existing sleep disorders were also excluded. All patients underwent a comprehensive ENT evaluation prior to undergoing bariatric surgery, evaluating for any possible need for surgical intervention.

### 2.2. Polysomnography and Anthropometry

Overnight PSG was performed by a blinded, registered sleep technician using the Alice PDx system (Respironics Inc., Murrysville, PA, USA). Polysomnography data were reviewed by at least two sleep technicians who were blinded to the patient’s clinical status. Participants were classified as having OSA based on their AHI scores, following the American Academy of Sleep Medicine (AASM) 2012 guidelines [17].

A detailed medical history was elicited and physical examination conducted along with anthropometric measurements such as body weight, height, and neck circumference. BMI was calculated using the formula; BMI = weight (in kilograms) ÷ [height (in meters)]^2^ for all participants. Following this process, patients with OSA underwent a non-contrast MRI scan of the upper airway to assess relevant anatomical parameters. Data were systematically recorded in a Microsoft excel spreadsheet.

### 2.3. Non-Contrast MRI

**Figure 1:**
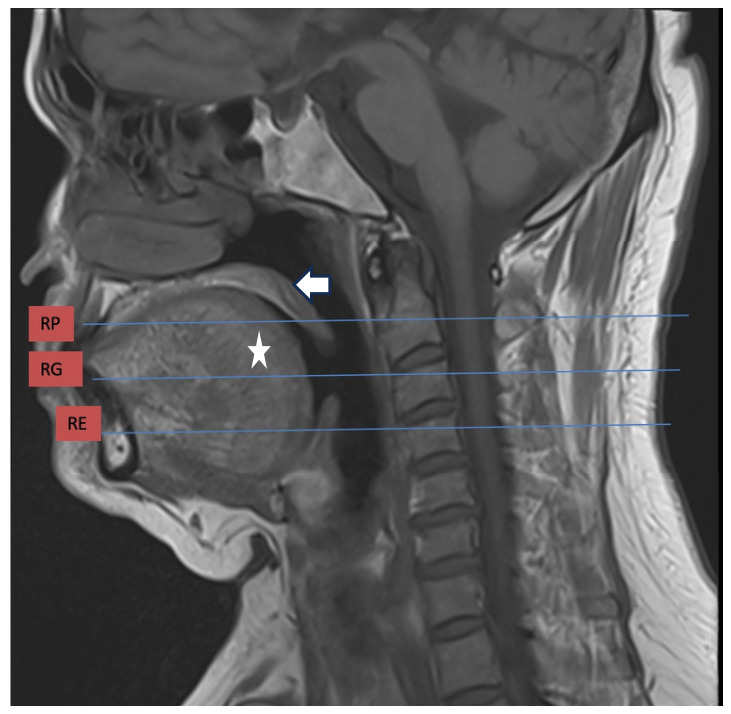
Sagittal T1W image showing tongue (*) and soft palate (arrow). RP: retropalatal, RG: retroglossal, RE: retro-epiglottal planes. The blue lines represent the respective planes.

Non-contrast MRI was conducted as per the same protocol used in a prior study from our center on a 1.5 T (T) MRI machine [Siemens Magentom Aera 1.5T (Erlangen, Germany)] with the patient lying awake, without any head and neck movements and avoiding swallowing, in the supine position in the Frankfurt plane [18]. The measurements were taken during normal respiration while breathing through the nose. The three axial planes were denoted as retro-palatal plane (at the mid-point of the soft palate), epiglottal plane (at the mid-point of the epiglottis), and retro-glossal plane (at the mid-point of the tip of the epiglottis and tip of the soft palate) (see Figure 1). The measurements were performed by an experienced radiologist with more than ten years of experience and blinded to the clinical status of the patient. The following MRI sequences were used:

1. Axial T1 spin-echo sequence with 3 mm slice thickness
2. Sagittal T1 spin-echo sequence with 5 mm slice thickness (Figure 1)
3. Axial T2 Dixon sequence with 3 mm slice thickness

**Figure 2:**
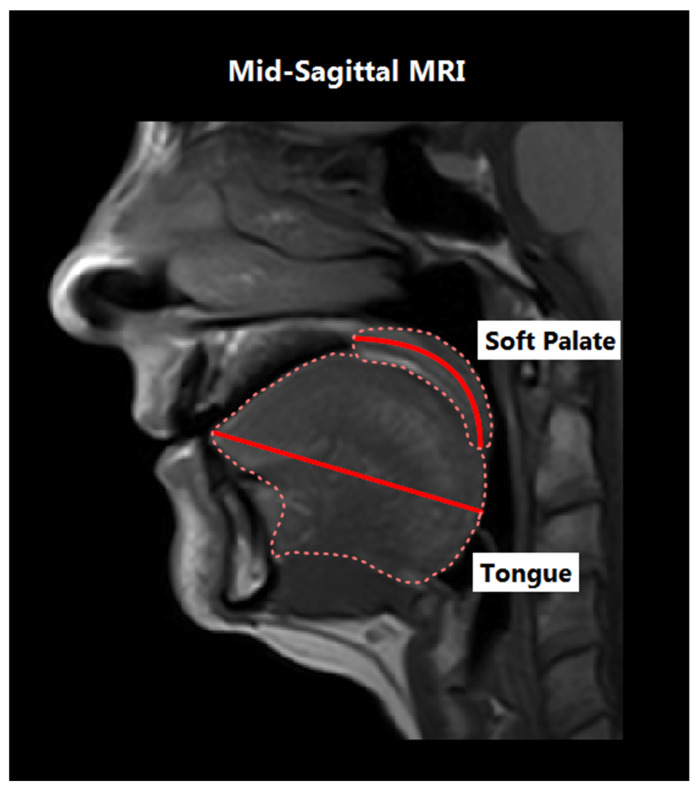
Soft Tissue Segmentation. Axial T1 spin-echo MRI slice demonstrating volumetric segmentation tongue, soft palate, medial pterygoids, parapharyngeal fat pads, lateral pharyngeal walls, and airway. Segmentations were performed using a combination of manual and semi-automated techniques.

The following parameters were assessed: Tongue volume, lateral pharyngeal wall volume, craniocaudal tongue length, and craniocaudal soft palate length were assessed in all patients. Tongue length constitutes the major axis, defined as the direct span between the most anterior and posterior points on midsagittal cross-section. Soft Palate length was acquired as a curved line following the course of the structure from its origin at the hard palate to the distal end of the uvula. Lengths of the tongue and soft palate were measured on the sagittal midline. (Figure 2).

**Figure 3:**
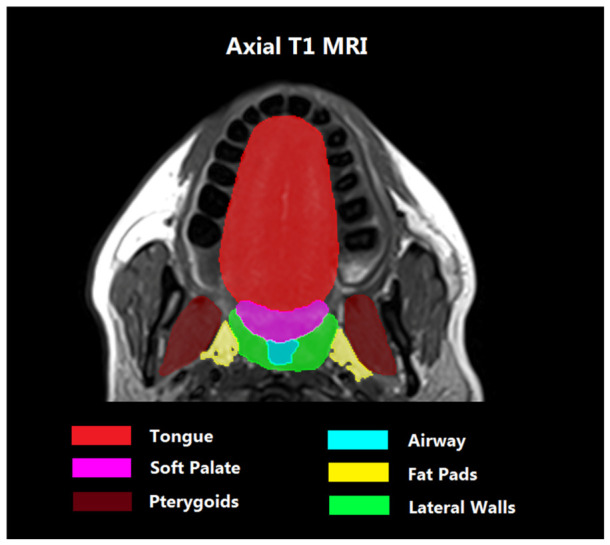
Soft Tissue Lengths. Mid-sagittal T1 MRI demonstrating measurement of tongue and soft palate length. Tongue length was acquired along the major axis, defined as the span between the most anterior and posterior points of the structure. Soft palate length was acquired along the curvilinear distance between the posterior nasal spine and distal end of uvula.

Volumetric measures of the airway, tongue, soft palate, medial pterygoids, lateral pharyngeal walls, and parapharyngeal fat pads were acquired using manual and semi-automated segmentation methods with Amira software (ThermoFisher Scientific, Waltham, MA, USA). Volumetric measures were captured using Axial T1 Spin-Echo MRI. (Figure 3).

**Figure 4:**
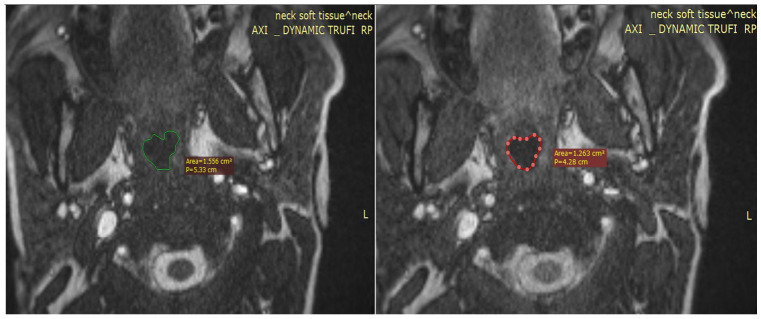
Axial dynamic imaging at retropalatal level showing respiratory variation in cross-sectional area. Green and red circles represent the airway cross sectional area.

**Figure 5:**
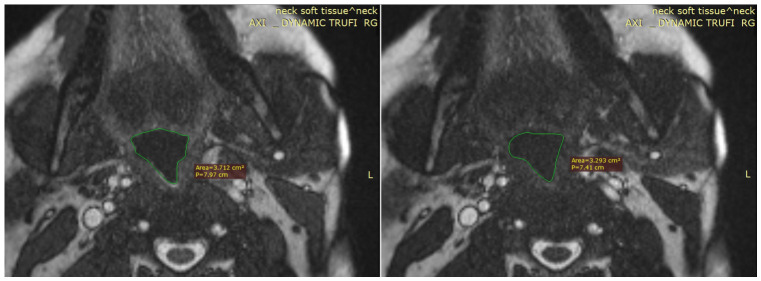
Axial dynamic imaging at retroglossal level showing respiratory variation in cross-sectional area. Green circles represent the airway cross sectional area.

In addition to volumetric measures of the airway, cross-sectional areas were acquired at the points of narrowest caliber in the retropalatal, retroglossal, and retroepiglottal regions. Additionally, average cross-sectional areas were calculated for these regions and for the airway as a whole. The area calculations are shown in (Figure 4 and Figure 5).

### 2.4. Statistical Analysis

STATA version 18.0 was used to perform the statistical analyses. Quantitative variables were summarized as median (Interquartile range, IQR). Wilcoxon signed-rank test (non-parametric test) was used to compare quantitative variables between baseline and post-operative groups for the small sample size (*n* = 10). Spearman correlation coefficients were calculated to estimate the relationship between percentage change in AHI, body weight and upper airway parameters. *p*-value < 0.05 was considered statistical significance. A *p*-value 0.005 (0.05/10) was taken to evaluate the significance of the correlations to account for multiple comparisons.

## 3. Results

Ten patients with OSA were followed up for 6 months after they underwent bariatric surgery. Mean age of the patients was 37.3 ± 11.2 years, with an equal number of males and females. At baseline, the patients had a mean BMI of 48.5 ± 5.8 kg/m^2^, indicating class 3 obesity.

### 3.1. Polysomnography and Anthropometry Parameters

Patients who underwent bariatric surgery demonstrated significant reductions in weight and BMI. The median weight decreased from 130 (IQR: 113–153) kg at baseline to 107.5 (IQR: 88.8–142) kg post-surgery (*p* = 0.002). Similarly, the BMI reduced from 46.4 (IQR: 44.8–52.3) kg/m^2^ to 39.0 (IQR: 35.6–44.5) kg/m^2^ (*p* = 0.002). These improvements in body composition were associated with significant reduction in the severity of OSA, as measured by AHI. The AHI showed a marked reduction from a median of 20.30/hour (16–52) at baseline to 12.80/hour (5.7–22.2) after surgery (*p* = 0.001) (Table 1).

### 3.2. Change in Upper Airway MRI Parameters

The upper airway anatomy showed notable reductions in pharyngeal soft tissue volumes. The median volumes of the soft palate [7490.6 mm^3^ (IQR: 6813.4–11,943.5) to 7030.8 (IQR: 6347.5–10,990.8), *p* = 0.005], pterygoid [14,127.3 (IQR: 11,329.7–15,382.9) mm^3^ to 13,023.4 (IQR: 10,552.2–14,000.5) mm^3^; *p* = 0.007)], tongue fat proportion [0.4% (IQR: 0.3–0.6%) to 0.3 (IQR: 0.2–0.4); *p* =0.02)], and para-pharyngeal fat pad volume [9556.9 (IQR: 7044.9–12,029.8) mm^3^ to 8251.6 (IQR: 5249.7–9143.5)] mm^3^; *p* = 0.009)] decreased significantly post six months of bariatric surgery. Overall, the total soft tissue volume (Table 2) decreased from 140,694.5 (IQR: 106,101.3–169,639.4) mm^3^ to 131,128.5 (IQR:104,362.9–157,168.4) mm^3^ (*p* = 0.02). Tongue size also decreased [83,977.3 (62,222.4–102,888.6) mm^3^ to 80,819.0 (IQR: 65,152.1–95,570) mm^3^] but this was not statistically significant, likely due to limited sample size.

Airway volume measurements did not reveal significant changes post-surgery. All airway volumes increased post bariatric surgery but did not attain statistical significance, likely due to limited sample size. Total airway volume increased from 6270.6 (IQR: 5655.4–8132) mm^3^ to 8322.0 (IQR: 4644.5–9169.6) mm^3^ (*p* = 0.88). Similarly, retropalatal [2253.8 (IQR: 1737.8–3056.3) mm^3^ to 2560.1 (IQR: 1650.8–3268.5) mm^3^, *p* = 0.72], and retroglossal [1194.5 (IQR: 935.9–1612.9) mm^3^ to 1691.7 (IQR: 1050.6–2344) mm^3^, *p* = 0.44)] airway volumes also increased but did not show statistical significance, possibly due to limited sample size.

### 3.3. Correlation Between Weight, AHI and Airway Parameters

There was no significant correlation between the percentage change in AHI and the percentage change in body weight (ρ = 0.28, *p* = 0.43) or BMI (ρ = 0.31, *p* = 0.38). Similarly, no significant correlations were observed between changes in dimensions of upper airway parameters and changes in body weight or AHI (Table 3), although the limited sample size might have resulted in the absence of significant correlation. Although there was no significant correlation between percentage change in body weight and ESS (ρ = 0.33, *p* = 0.35), statistically significant correlation was noted with waist circumference (ρ = 0.84, *p* = 0.002) and neck circumference (ρ = 0.758, *p* = 0.001), significant at Bonferroni threshold of 0.005.

## 4. Discussion

Although limited by numbers, study showed a significant reduction in anthropometric parameters such as weight and BMI and non-significant reduction in neck and waist circumference in patients with OSA and obesity who underwent bariatric surgery. Weight loss resulting from bariatric surgery was associated with a significant reduction in AHI (severity of OSA) after surgery, although the treatment response was heterogenous across individuals. Previous studies have shown improvement in AHI after bariatric surgery, likely to be primarily due to reduction in obesity-related upper airway load and by enhancing airway patency during sleep [19,20,21,22]. The heterogeneity in response is similar to published literature which suggests an overall decrease in severity without uniformly curing OSA; reported remission rates ranging from 26 to 76% [23,24].

The study findings also demonstrated a significant reduction in volumes of the soft palate, pterygoid muscles, para-pharyngeal fat pads, tongue fat fraction, and total soft tissue volume. These findings underscore the relationship between weight reduction and soft tissue composition of the upper airway. These study results show a preferential decrease in para-pharyngeal fat depots and certain soft tissue structures with weight reduction after bariatric surgery, which may be key contributors to upper airway collapsibility. Previous imaging studies have shown that obese individuals with OSA may have enlarged pharyngeal soft tissues, including excess soft-tissue volume, tongue volume, parapharyngeal fat, lateral wall volume, which predispose to upper airway obstruction even with a normal airway anatomy [25,26,27,28]. Among the upper airway soft tissue compartments, tongue fat has the strongest mechanistic link to OSA severity, with enlargement of tongue being associated with reduced lumen size and increased collapsibility. Further, fat deposition in parapharyngeal space and lateral pharyngeal walls have also been associated with dynamic airway collapse due to increase in pharyngeal wall compliance. Although the study used this segmentation strategy, differentiation of individual fat depots was not performed and hence results should be interpreted cautiously without attributing a direct causal relationship.

Interestingly, despite these reductions in soft tissue volumes, no statistically significant increase in the airway lumen size and cross-sectional areas were noted in this pilot cohort, although estimates are imprecise and should be interpreted cautiously, given the small sample size of the study. Further, OSA anatomy is not uniform across the airway, with specific regions like velopharynx likely to be the most important sites of obstruction. Global airway measurements alone as done in this study may miss regional changes at specific levels which may occur after surgery or weight loss. Further, awake changes on MRI assessed in this study may not fully capture the dynamic airway behavior occurring during sleep in patients with OSA.

OSA is increasingly recognized as a heterogenous disorder involving both anatomical and non-anatomical pathophysiological traits; the latter including arousal threshold, passive critical closing pressure of the upper airway, loop gain and responsiveness of upper airway dilator muscles [29]. The extent to which weight loss results in improvement in AHI is influenced by these non-anatomical factors as well. So, while the present study examined the anatomical factors, the impact of neural and physiological factors needs to be evaluated.

Exploratory analysis found no significant correlation between percentage change in body weight and percentage changes in ESS, and AHI. However, a statistically significant positive correlation was observed between the percentage change in weight loss with that of change in neck and waist circumference. Interestingly, no significant correlation was found between percentage changes in weight, AHI, and upper airway parameters. Notably, despite improvement in BMI and AHI post-surgery, the study cohort remained obese post-operatively (median BMI 39 kg/m^2^) with residual OSA (median AHI 12.8/h) indicating residual OSA. Although PSG parameters represented improvement, it did not fulfill the commonly used Sher criteria to define surgical success (defined as at least 50% reduction in AHI/RDI with a post-operative AHI/RDI below 20 [30]). Thus, residual obesity and limited normalization of OSA severity may have limited the magnitude of measurable airway remodeling and attenuated anatomical correlations in this cohort. Previous imaging studies in larger cohorts have reported mixed findings on the correlation between improvement in AHI and upper airway structural changes following weight loss. Current literature from MRI-based studies have supported a close relationship between obesity, tongue adiposity and OSA. Tongue fat has been previously shown to be increased in obese individuals with OSA compared to obese controls without OSA, implicating tongue fat as a potentially important obesity-related anatomic trait [7]. Shigeta et al. had, however, reported no significant correlation between AHI and tongue volume in Japanese male OSA patients, similar to the results in this study cohort [31]. Ethnic variation in craniofacial anatomy may partly explain the differences between Asian and Western populations. Asian patients with OSA have been shown to have greater craniofacial bony restriction including shorter mandibular length and smaller retropalatal airway dimensions restricting airway expansion and predisposing to airway collapse, despite lower levels of obesity [11,15]. Likewise, Ahn et al. did not find significant association between change in tongue volume or posterior airway space and change in AHI in Korean patients with OSA [32]. In a previous study involving 60 individuals undergoing weight loss interventions, waist circumference was identified as a mediator between weight loss and AHI reduction using mediation analysis [33]. However, the small sample size of the present study did not permit a similar mediation analysis. Although the tongue fat fraction decreased post bariatric surgery in the present study, directionally consistent with present literature, the persistence of post-operative obesity and the small sample size may have limited the magnitude and precision of any observed association.

This pilot study, to the best of our knowledge, represents one of the few studies evaluating the effects of bariatric surgery-induced weight loss on upper airway anatomy in Asian population. Upper airway morphology was assessed through MRI which provides superior soft tissue contrast and more accurate quantification of airway soft tissue structures compared to conventional cephalometric radiography. Further exploratory analyses were performed to evaluate the potential association between sleep parameters, upper airway measurements, and clinical characteristics of the study participants.

## 5. Limitations

The study has quite a few limitations. Firstly, the small sample size limited statistical power and precluded multivariable analyses, resulting in imprecise estimates and limited the ability to adjust for potential confounders. The absence of statistically significant difference in size of the airway lumen and cross-sectional areas across upper airway despite reduction in soft tissues volumes should be interpreted in the light of the small sample size of this pilot study. The study also did not assess region-specific changes in airway anatomy and fat deposition across specific areas. Secondly, the absence of a control group restricts casual interference as comparison with patients managed through non-surgical weight loss strategies or other interventions like uvulopalatopharyngoplasty were not possible. Also, the study cohort included relatively few patients with mild OSA which limits generalizability of the findings across the entire spectrum of disease severity. Thirdly, MRI imaging was performed only in the awake state, which may not fully capture the dynamic airway behavior contributing to OSA pathophysiology. Furthermore, bony craniofacial parameters were not systematically evaluated and analyzed as a part of the study, and mediation analysis could not be performed due to the small sample size. Standardized anatomical findings including structured nasal evaluation and evaluation of nasopharynx and tongue were not included in the overall analysis. Future prospective studies with larger cohorts, control groups which include those managed by non-surgical weight loss strategies, and alternative interventions, including uvulopalatopharyngoplasty, are needed for establishing causal interference and imaging approaches that better capture dynamic airway behavior during sleep which may better elucidate the mechanisms linking weight loss and improvements in OSA.

## 6. Conclusions

Weight loss following bariatric surgery was associated with reductions in the volumes of several pharyngeal soft tissue structures in this cohort. However, no significant correlation was noted between changes in upper airway anatomical parameters and changes in AHI. However, there was reduction in pharyngeal soft tissue volumes, parapharyngeal fat pad volume, tongue fat fraction and a trend towards increase in airway volume measurements. These findings should, however, be interpreted in the context of limited sample size of this pilot study and need validation in larger cohorts. Further, the limited data from the study seems to suggest that improvements in OSA following weight loss may not be explained entirely by measurable changes in static upper airway anatomical parameters, and non-anatomical mechanisms may also contribute to disease improvement, which needs further evaluation in future studies.

## Figures and Tables

**Table 1:** Polysomnography and anthropometry parameters pre- and post-surgery in OSA patients.

Parameters	Baseline (*n* = 10)	Post-Surgery (*n* = 10)	*p* Value
Weight (kg) Median (IQR)	130 (113–153)	107.5 (88.8–142)	0.002 *
Body mass index (kg/m^2^) Median (IQR)	46.4 (44.8–52.3)	39.0 (35.6–44.5)	0.002 *
Waist circumference (cm) Median (IQR)	131 (124–137)	123 (110–124)	0.36
Neck circumference (cm) (Mean ± SD)	42.7 ± 3.6	39.1 ± 5.0	0.083
Apnea–hypopnea index Median (IQR)	20.30 (16–52)	12.80 (5.7–22.2)	0.0011 *
Epworth sleepiness scale	10.0 (6.0–10.0)	4.0 (2.0–7.0)	0.016 *

* Indicates statistical significance (*p* < 0.05).

**Table 2:** Magnetic resonance imaging-based upper airway parameters before and after bariatric surgery. (*n* = 10).

Parameters	Baseline Median (IQR)	Post-Surgery Median (IQR)	*p* Value
Soft palate volume (mm^3^)	7490.6 (6813.4–11,943.5)	7030.8 (6347.5–10,990.8)	0.005 *
Tongue volume (mm^3^)	83,977.3 (62,222.4–102,888.6)	80,819.0 (65,152.1–95,570)	0.14
Pterygoid volume (mm^3^)	14,127.3 (11,329.7–15,382.9)	13,023.4 (10,552.2–14,000.5)	0.007 *
Para-pharyngeal fat pad volume (mm^3^)	9556.9 (7044.9–12,029.8)	8251.6 (5249.7–9143.5)	0.009 *
Retropalatal lateral walls volume (mm^3^)	11,979.7 (6624.5–19,020.5)	10,997.7 (6278.2–14,998.3)	0.39
Retroglossal lateral walls volume (mm^3^)	9793.1 (3940.5–12,269.1)	8770.9 (5092.4–16,176.8)	0.80
Tongue fat fraction (%)	0.4 (0.3–0.6)	0.3 (0.2–0.4)	0.005
Total soft tissue volumes (mm^3^)	140,694.5 (106,101.3–169,639.4)	131,128.5 (104,362.9–157,168.4)	0.02 *
Soft palate length (mm)	34.4 (33–39.8)	33.3 (31.2–37.8)	0.09
Tongue length (mm)	79.2 (70.4–82.2)	78.9 (70.5–82.7)	0.51
Retropalatal airway volume (mm^3^)	2253.8 (1737.8–3056.3)	2560.1 (1650.8–3268.5)	0.72
Retroglossal airway volume (mm^3^)	1194.5 (935.9–1612.9)	1691.7 (1050.6–2344)	0.44
Total airway volume (mm^3^)	6270.6 (5655.4–8132)	8322.0 (4644.5–9169.6)	0.88

* Indicates statistical signficance (*p* < 0.05).

**Table 3:** Correlation between percentage change in body weight, apnea–hypopnea index and upper airway parameters.

% Change in Upper Airway Parameters	% Change in Body Weight		% Change in Apnea–Hypopnea Index	
	ρ (95% CI)	*p* Value	ρ (95% CI)	*p* Value
Soft palate volume	−0.20 (−0.74, 0.49)	0.58	0.07 (−0.59, 0.67)	0.85
Tongue volume	0.35 (−0.36, 0.80)	0.33	−0.01 (−0.63, 0.63)	0.99
Pterygoid volume	0.28 (−0.42, 0.78)	0.43	−0.04 (−0.66, 0.60)	0.91
Para-pharyngeal fat pad volume	−0.05 (−0.66, 0.60)	0.88	−0.32 (−0.79, 0.39)	0.37
Total soft tissue volume	0.27 (−0.43, 0.77)	0.45	0.04 (−0.60, 0.66)	0.91
Retropalatal airway volume	−0.16 (−0.72, 0.52)	0.65	0.15 (−0.53, 0.71)	0.68
Retroglossal airway volume	−0.18 (−0.73, 0.51)	0.63	0.01 (−0.63, 0.63)	0.99
Total airway volume	0.12 (−0.56, 0.69)	0.75	0.30 (−0.41, 0.78)	0.40
Soft palate length	0.35 (−0.36, 0.80)	0.33	0.27 (−0.43, 0.77)	0.45
Tongue length	0.10 (−0.56, 0.69)	0.78	0.13 (−0.55, 0.70)	0.77
Tongue fat	0.41 (−0.30, 0.83)	0.24	0.09 (−0.57, 0.68)	0.80

%: percentage, ρ: Spearman’s correlation co-efficient. All *p*-values evaluated for statistical significance at *p* < 0.003.

## Data Availability

The datasets generated and/or analyzed during the current study are available from the corresponding author upon reasonable request.

## Abbreviation

OSA: Obstructive Sleep Apnea; AHI: Apnea–Hypopnea Index; MRI: Magnetic Resonance Imaging; PSG: Polysomnography; BMI: Body Mass Index; ESS: Epworth Sleepiness Scale; AASM: American Academy of Sleep Medicine; CPAP: Continuous Positive Airway Pressure; SD: Standard Deviation; T: Tesla (MRI magnetic field strength); T1W: T1-weighted imaging; T2W: T2-weighted imaging; CI: Confidence Interval; ρ (rho): Spearman’s Correlation Coefficient.
